# How does agricultural insurance affect income inequality within farmers? Based on survey data from Shandong Province, China

**DOI:** 10.1371/journal.pone.0337529

**Published:** 2025-12-22

**Authors:** Zongyun She, Le Sun, Yanmei Yuan, Shengwei Chen

**Affiliations:** 1 College of Economics and Management, Shandong Agricultural University, Tai’an, China; 2 Faculty of General Education, Taishan College of Science and Technology, Tai’an, China; 3 School of Economics, Shandong University of Technology, Zibo, China; Shandong University of Science and Technology, CHINA

## Abstract

To solidly promote common prosperity, the most arduous and heavy task still lies in rural areas. Agricultural insurance, as an important tool for supporting and benefiting agriculture, is of great significance in narrowing the income gap among farmers and achieving common prosperity in rural areas. This article uses survey data from 632 households in Shandong Province to empirically test the impact and mechanism of agricultural insurance on income inequality among farmers. It has been found that purchasing agricultural insurance can alleviate income inequality within farmers. This impact is achieved through three pathways: promoting the transfer of agricultural land, easing credit constraints, and optimizing labor resource allocation. Further research has found that purchasing agricultural insurance can significantly suppress income inequality among households in the dependency and stable periods, while having no impact on households in other stages of their life cycle. To promote common prosperity, this article believes that we should continue to promote the “expansion” of agricultural insurance, strengthen the coordination and cooperation between agricultural insurance and other agricultural support policies, and promote the differentiation of agricultural insurance products.

## 1. Introduction

The report of the 20th National Congress of the CPC clearly pointed out that “achieving common prosperity for all people is the essential requirement of Chinese path to modernization”, while the most arduous task of solidly promoting common prosperity is still in the countryside. At present, the income distribution gap among Chinese residents has been hovering at a high level [1,2]. Although the Chinese government has effectively reduced the income gap among residents through the implementation of targeted poverty alleviation and targeted poverty alleviation strategies, according to data from the National Bureau of Statistics of China, the Gini coefficient of China’s income in 2022 was 0.46, which is still at a high level, higher than Germany’s 0.29 and France’s 0.32 at the same time, and also higher than East Asian countries such as Japan and South Korea, exceeding the internationally recognized warning line of 0.4 [3]. However, the uneven distribution of internal income among rural residents in China is more prominent and continues to worsen. According to the China Statistical Yearbook, the per capita disposable income gap between high-income and low-income households in rural China has widened from 6.47 times in 2000 to 9.52 times in 2023. The continuous widening of income inequality not only affects social stability, reduces investment, and hinders economic growth [4], but also leads to an imbalance in the structure of rural labor force [5], a decrease in the happiness of rural residents [6], and excessive concentration of land production factors, which damages the efficiency of rural development. Therefore, for common prosperity, it is urgent to explore feasible paths to promote income growth for low-income groups and accelerate narrowing income gaps among farmers.

Agricultural insurance is an important agricultural risk management tool in China [7]. Since 2007, the Chinese government has continuously strengthened financial subsidies for agricultural insurance, improved food crop insurance protection, and encouraged the development of characteristic agricultural product insurance. The scale of agricultural insurance premiums has rapidly grown, with an average annual growth rate of 22%. By the end of 2024, the premium scale of China’s agricultural insurance has reached a new high, increasing to 152.1 billion yuan, providing risk protection for 147 million households with a total of over 5 trillion yuan. At the same time, the depth and density of agricultural insurance have shown a steady upward trend, reaching 1.66% and 942.67 yuan/person, respectively, playing an important role in ensuring the stable development of the agricultural industry and increasing farmers’ income.

However, in the context of the continuously widening income gap among rural residents, there is no research indicating whether the income-increasing effect of agricultural insurance varies among farmers with different income levels, that is, whether agricultural insurance can alleviate the problem of income inequality among farmers while increasing their absolute income. Therefore, this article intends to use micro survey data from 632 households in Shandong Province to examine the impact and mechanism path of agricultural insurance on the income gap among farmers from the perspective of factor allocation. This has important practical significance for helping achieve the goal of common prosperity in rural areas.

The possible marginal contribution of this article lies in: firstly, based on a micro perspective, by exploring the impact of purchasing agricultural insurance on internal income inequality among farmers from both theoretical and empirical perspectives, this article provides new evidence for agricultural insurance to promote income equality among farmers; secondly, by introducing the mechanism of factor allocation, and exploring the multiple mechanisms of the impact of agricultural insurance on income inequality within farmers from three dimensions of land transfer, credit acquisition, and labor allocation, this article enriches the existing theoretical framework; thirdly, by demonstrating the heterogeneous impact of farmers purchasing agricultural insurance on income inequality at different stages of the family life cycle, this article is conducive to further clarifying the effect of agricultural insurance on changes in household income.

## 2. Literature review

There have been rich discussions on the income effects of agricultural insurance in existing studies, and the literature related to this article mainly focuses on two aspects: first, the impact of agricultural insurance on farmers’ absolute income; second, the impact of agricultural insurance on income inequality.

In terms of the impact of agricultural insurance on farmers’ income, the risk diversification and loss compensation functions of agricultural insurance have a smoothing effect on agricultural income fluctuations [8,9]. As Leathers et al. (1991) [10] found in their study of agricultural insurance in the United States, when the payout amount of agricultural insurance increased by $1, farmers’ income increased by $1.03; Enjolras et al. (2012) [11] found through empirical analysis of data from 9555 farms in France and Italy that agricultural insurance can reduce fluctuations in farmers’ income and plays an important role in stabilizing farmers’ income. In addition, after purchasing agricultural insurance, farmers will have incentive effects, which encourage them to optimize the allocation of production factors [12], expand their business scale, increase production investment [13], and improve their professional planting level [14], thereby further enhancing their income level. The higher the level of agricultural insurance protection, the greater the effect on improving farmers’ income level [15]. However, some scholars have pointed out that agricultural insurance has a threshold effect on farmers’ income, and only when the income exceeds a certain threshold can agricultural insurance play a role in increasing farmers’ income [16].

Regarding the impact of agricultural insurance on income inequality, agricultural insurance has the function of income redistribution and is an important means of government fiscal transfer payments [17,18]. The higher the subsidy ratio and protection level of agricultural insurance, the more helpful it is to curb income inequality among farmers [19]. In addition, the coordinated development of agricultural support policies such as agricultural insurance, agricultural credit [20], and agricultural subsidies [21] also impacts the income gap among farmers. In terms of the impact of agricultural insurance on the income gap between urban and rural residents, existing research suggests that agricultural insurance can not only motivate farmers to engage in specialized and large-scale operations [22], thereby increasing their agricultural income, but also enhance their non-agricultural income by strengthening their career selection mechanism and optimizing the allocation of labor resources [23]. However, as a policy tool to support and benefit agriculture, agricultural insurance cannot have a significant impact on the income level of urban residents [24]. Therefore, existing research suggests that agricultural insurance has the effect of narrowing the income gap between urban and rural residents.

Overall, existing research has specific reference value for this article, but it mainly focuses on the impact of agricultural insurance on the urban-rural income gap. Few studies systematically explore the impact of agricultural insurance on income inequality among farmers from a micro perspective, and the impact mechanism is not precise. Therefore, this article will empirically test the impact and mechanism of agricultural insurance on income inequality among farmers, and further examine the heterogeneity of different family types based on the theory of family life cycle, providing theoretical support for the efficient utilization of the co-prosperity effect of agricultural insurance.

## 3. Theoretical analysis and research hypotheses

### 3.1 The impact of agricultural insurance on income inequality among farmers

Agricultural risk is an important factor affecting farmers’ income. According to the theory of risk and uncertainty, risk-averse farmers will make different decisions due to differences in risk exposure, further widening the income gap. Specifically, high-income farmers have higher capital accumulation and can quickly restore agricultural production after risks occur. This high risk tolerance makes the production decision-making standards of high-income farmers closer to “maximizing expected utility”, promoting their efficient input and use of production factors. Low-income farmers, on the other hand, lack risk-buffering assets and have a high degree of loss aversion. They generally do not rashly increase investment in production factors such as land and capital, but adopt a “low-risk, low return” production strategy. Therefore, the marginal negative impact of risk shocks on low-income farmers is much higher than that on high-income farmers, amplifying the differences in farmers’ capital endowments and increasing income inequality. Agricultural insurance can transform uncertain significant losses of farmers into a definite and small expenditure, enabling agricultural producers to achieve higher agricultural risk protection at lower costs [12], and curbing the effect of agricultural risks exacerbating income inequality. In addition, policy-based agricultural insurance achieves income redistribution through premium subsidies [18] and compensates insured farmers for agricultural production losses after risks occur [24]. The higher payout ratio of policy-based agricultural insurance further increases the income level of insured farmers [25], which motivates farmers to increase their input of production factors. This incentive effect will have a higher marginal contribution and positive externality for low-income farmers, narrowing the income gap between farmers.

Based on the above analysis, this article proposes hypothesis 1: Purchasing agricultural insurance can effectively reduce income inequality among farmers.

### 3.2 The impact mechanism of agricultural insurance on income inequality among farmers

**Fig 1:**
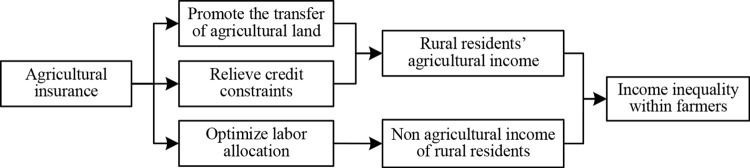
The impact mechanism of agricultural insurance on income inequality among farmers.

The theory of endowment of production factors points out that different entities have endowment differences in land, capital, and labor factors, which determine their behavioral decisions. This theory also has good explanatory power at the micro level of farmers, viewing them as micro “economic entities” whose production decisions and income levels are endogenous to their land, capital, and labor inputs. The previous analysis shows that differences in agricultural risk exposure and farmers’ production decisions have led to income inequality. Agricultural insurance, as an important tool to ensure agricultural production, can diversify agricultural risks, increase farmers’ income expectations, and thus change farmers’ factor inputs [13,26], ultimately affecting farmers’ income levels. Therefore, this article will analyze the factor allocation mechanism of agricultural insurance in alleviating income inequality among farmers from three aspects: land transfer (land), credit acquisition (capital), and labor resource allocation (labor). The logical framework diagram is shown in Fig 1.

(1) Agricultural insurance, land transfer, and income inequality among farmers

After purchasing agricultural insurance, farmers can effectively cope with the negative impact of natural disasters on agricultural production [19], ensure relatively stable expected income in agricultural management, and alleviate constraints on labor, capital, technology and other factors in agricultural production, ultimately achieving the effect of incentivizing farmers to transfer land and expand their business scale [22,27]. At the same time, with the continuous improvement of the risk protection level of agricultural insurance, the motivation of farmers to transfer to land and expand their business scale is also constantly increasing. After transferring to farmland, farmers will further increase agricultural production profits and increase agricultural income due to the marginal profit of operating farmland being greater than net rent. Compared to high-income farming households, low-income farming households generally rely on their own labor for intensive farming due to limited capital accumulation, resulting in lower input costs and higher marginal output of agricultural land. Therefore, the net income growth rate of agricultural production after low-income households transfer to farmland is higher than that of high-income households. At the same time, due to the small scale of agricultural land and the seasonal characteristics of agricultural production, low-income farmers who adopt small-scale agricultural production and management methods generally have part-time jobs [28], further increasing non-agricultural income. However, high-income farming households tend to allocate more production resources to agricultural production due to large-scale production, which often limits the growth of non-agricultural income. Therefore, transferring land is beneficial for reducing income inequality among farmers.

(2) Agricultural insurance, credit access, and income inequality among farmers

There is a significant difference in credit availability between high-income and low-income farmers. High-income farmers have a high capital endowment and can obtain loans in various forms, such as mortgages and guarantees, to maintain stable agricultural production. However, low-income farmers lack suitable collateral, have low access to credit, and find it difficult to expand production. In a disaster, it is not easy to maintain simple production. With the risk protection of agricultural insurance being incorporated into the risk screening mechanism of lending institutions, the credit enhancement function of agricultural insurance is gradually emerging. On the one hand, agricultural insurance stabilizes farmers’ income, improves their debt repayment ability, and is beneficial for farmers to obtain credit loans. With the improvement of the level of protection, the credit enhancement effect of agricultural insurance and the ability of farmers to obtain loans will also be further strengthened [13].On the other hand, with the support of agricultural policies such as “bank insurance interaction”, agricultural insurance can be used as collateral for farmers’ credit, solving the problem of credit allocation in rural areas [29], and significantly improving farmers’ ability to obtain mortgage loans [30]. It is worth noting that the credit enhancement function of agricultural insurance is also limited, usually only 30% −90% of the insurance amount. This type of small-scale loan may seem insignificant for high-income farmers with high capital endowments. However, it can alleviate the financial difficulties of low-income farmers’ production capital turnover. Therefore, it usually manifests as low-income farmers being more willing to obtain loans through agricultural insurance to increase their creditworthiness. In addition, alleviating credit constraints on low-income farmers can motivate them to expand their production scale and improve agricultural production efficiency gradually [13], thereby increasing agricultural operating income, narrowing the income gap with high-income farmers, and alleviating internal income inequality among farmers.

(3) Agricultural insurance, labor allocation, and income inequality among farmers

According to the previous analysis, purchasing agricultural insurance will incentivize farmers’ agricultural production. This is because after the production risks of farmers are diversified, their expected income increases accordingly, which promotes the shift of farmers’ production activities from risk avoidance to risk neutrality, effectively enhancing their adoption behavior of new seeds and technologies [31]. Adopting new technologies not only improves the unit output level but also further enhances output efficiency, thereby saving more labor and promoting its investment in non-agricultural sectors, optimizing labor resource allocation. Studies have shown that the free flow of labor can balance efficiency and balance, and there are specific differences in the income-increasing effects on farmers with different income levels [32]. Low-income farmers’ original dependence on land is strong, and they invest more in agricultural labor. After agricultural insurance generates surplus labor resources by improving production efficiency, low-income farmers have more opportunities to invest their labor in non-agricultural work, thereby improving their income level. For high-income farmers, there are two situations: one is high-income farmers with a high proportion of non-agricultural income, and the other is that the ability of agricultural insurance to release their labor is limited. The other is high-income farmers engaged in large-scale agricultural operations, who rely heavily on agricultural operating income and will continue to invest their labor in low-productivity agricultural sectors even after releasing their labor. From this perspective, the effect of agricultural insurance on promoting labor allocation among low-income farmers is significantly higher than that of high-income farmers, which is conducive to reducing income inequality among farmers.

Based on the above analysis, this article proposes the following hypothesis:

Hypothesis 2-1: Agricultural insurance can incentivize farmers to transfer to land, thereby reducing income inequality.
Hypothesis 2-2: Agricultural insurance can alleviate credit constraints on farmers, thereby reducing income inequality.
Hypothesis 2-3: Agricultural insurance can optimize the allocation of labor resources, thereby reducing income inequality.

## 4. Materials and research methods

### 4.1 Data sources and basic characteristics of samples

The data in this article is sourced from a survey conducted by the research team in 16 cities in Shandong Province in January 2025, with the theme of “Agricultural Production Behavior of Farmers”. The survey was conducted through a combination of household interviews and online distribution of survey questionnaires. The questionnaire mainly included personal and family information of farmers, agricultural production situation, agricultural production risk situation, and insurance characteristics. A total of 700 questionnaires were distributed in this research activity, and 632 valid questionnaires were obtained, with a questionnaire effectiveness rate of 90.3%. This study involved only the analysis of fully anonymized datasets. In accordance with the Ethical Review Measures for Life Sciences and Medical Research Involving Humans issued by the National Health Commission of China and three other ministries, the ethics committee granted a waiver of written informed consent for this study. Nonetheless, all participants were fully informed at the initial data collection stage about the research purposes, potential data usage, confidentiality measures, and their right to withdraw at any time without penalty. Verbal informed consent was obtained from all participants and documented via detailed notes in the research log.

From the basic characteristics of the sample farmers, the sample with household heads aged between 36 and 65 accounted for 69.62% of the total sample; The education level of the household head is generally at the junior high school level or below; The population size of the sample households is mostly between 3 and 6 people; More than 70% of the sample are farmers engaged in full-time and part-time farming work; The land management scale of the surveyed households is generally below 10 acres; 79.9% of households have an annual total income below one hundred thousand yuan, while less than 10% have an annual income exceeding one hundred and fifty thousand yuan. The basic characteristics of the sample farmers are shown in Table 1.

**Table 1:** Basic characteristics of sample farmers.

Variable	Category	Frequency	Proportion(%)
Head of household age (years)	[18, 35]	48	7.59
	[36, 50]	204	32.28
	[51, 65]	236	37.34
	[66, 89]	144	22.78
Year of education for the head of household (in years)	[0, 6]	296	46.84
	[7, 9]	216	34.18
	[10, 12]	64	10.13
	[13, 16]	56	8.86
Total household population (person)	[1,2]	44	6.96
	[3, 4]	276	43.67
	[5, 6]	269	42.56
	[7, 10]	44	6.96
Employment status of household head	Full duty farmer	232	36.71
	Non agricultural employment	120	18.99
	Part-time farmers	224	35.44
	unemployed	56	8.86
Land management scale (mu)	(0, 5]	344	54.43
	(5, 10]	172	27.22
	(10, 20]	64	10.13
	(20, 100]	52	8.23
Annual total household income (ten thousand yuan)	[0, 5]	240	37.97
	(5, 10]	265	41.93
	(10, 15]	64	10.13
	(15, 20]	63	9.97

### 4.2 Variable selection

1 Explained variable: income inequality among farmers. Regarding the measurement of income inequality, this article draws on the research of Hua and Pan (2024) [33] and Su et al. (2024) [34]. It uses the Kakwani Relative Deprivation Index to measure the degree of income inequality among farmers based on their total household income. Among them, the total income of rural households consists of four parts: operating income, wage income, property income, and transfer payment income. The specific calculation formula for Kakwani’s relative deprivation index is as follows:

$$\begin{array}{c} \text{Kakwani}=\frac{\text{1}}{n\mu_{x}}\sum_{i=k+\text{1}}^{n}\left(x_{i}-x_{k}\right)=\gamma_{x_{k}}^{+}\left[\frac{\mu_{x_{k}}^{+}-x_{k}}{\mu_{x}}\right] \end{array}$$ (1)

In the above equation, *X* represents the group of farmers with a total sample size of *n*, and the corresponding income vector is X=(*x1*, *x2*,..., *xn*), arranged in ascending order of income. The deprivation suffered by farmer *xk* is recorded as Kakwani, which $\mu_{x_{k}}^{+}$ is the average income of the sample with income exceeding *xk* in *X*, the percentage of the sample with $\gamma_{{\text{x}}_{\text{k}}}^{+}$ income exceeding *xk* in the total $\mu_{x}$ sample *X*, and the average income of the total sample *X*. The value range of this index is between 0 and 1. The larger the value, the more severe the relative deprivation of social resources suffered by farmers, and the higher the degree of income inequality among farmers.

2 Core explanatory variable: whether farmers have purchased agricultural insurance. This article uses “whether to purchase agricultural insurance in 2024”to characterize farmers’ agricultural insurance purchasing behavior. According to research data, the agricultural insurance coverage rate for farmers in the entire sample is 40.35%.
3 Mediating variables: based on the theoretical analysis in the previous text, this article takes the allocation of production factors as the mechanism variable, and specifically tests the impact mechanism of agricultural insurance on farmers’ income inequality from three dimensions: land transfer, credit acquisition, and non-agricultural employment. Among them, as for (1) farmland transfer, this article uses the phrase ‘Did you transfer land in 2024?’ to characterize the agricultural land transfer behavior of rural households; (2) credit acquisition, this artile measures the credit situation of farmers by asking “Have you applied for loans from financial institutions in 2024? ” and assign a value of 0 to the farmer’s “expected loan amount not obtained”; Assign a value of 1 to ‘obtain expected loan amount’. (3) cabor force allocation, this article draws on the research of Wu et al. (2025) [35] and uses the ratio of the number of people engaged in non-agricultural labor in a household to the total household population as a proxy variable for non-agricultural employment in rural households.
4 Controlling variables: to controlling the impact of other variables on income inequality among farmers and prevent estimation errors caused by omitted variables, this article selects control variables from three aspects: individual characteristics of household heads, family characteristics, and agricultural production and operation characteristics. In terms of individual characteristics of household heads, five control variables are selected: gender, age, education level, political status, and physical health status; in terms of household characteristics, it specifically includes the number of household members and insurance coverage; in terms of agricultural production characteristics, four variables were controlled for: whether one had received agricultural technology training, whether one had joined an agricultural cooperative, land management area, and degree of land fragmentation. The meanings and descriptive statistics of the above variables are shown in Table 2.

**Table 2:** Descriptive statistics of each variable.

Variable type	Variable	symbol	Variable meaning	mean value	standard deviation
Explained Variable	Income inequality	Kawani	Calculated based on Kawani’s individual income relative deprivation index	0.3647	0.2488
Core explanatory variables	Whether to participate in agriculture insurance	*insur*	No = 0; Yes = 1	0.4035	0.4910
Controlling variable	gender	*gender*	Female = 0; Male = 1	0.5791	0.4941
	age	*age*	The actual age of the household head	55.3108	15.3583
	Educational level	*edu*	Not attending school = 1; Primary school = 2; Junior high school = 3; High school = 4; College and above=5	2.6709	1.1115
	Political status	*poli*	Ordinary people = 1; Communist Party member = 2; Communist Youth League members = 3; Democratic Party = 4	1.1203	0.3958
	Physical health	*heal*	Very poor = 1; Comparatively poor = 2; Generally = 3; Better = 4; Very good = 5	3.7342	0.8229
	Number of household members	*labor*	Total number of family members	4.6625	1.6197
	insurance awareness	*inawar*	Whether to purchase other insurance: No = 0; Yes = 1	0.8291	0.3767
	Do you want to join an agricultural cooperative	*coop*	No = 0; Yes = 1	0.7785	0.4156
	Do you accept agriculture Technical training	*train*	No = 0; Yes = 1	0.3038	0.4603
	land fragmentation degree	*frag*	Land management area/number of land management blocks	4.0887	7.8121
	Land management area	*land*	Actual land area operated by farmers in 2024	12.5504	22.6759
Mechanism variables	Transfer of agricultural land	*tran*	Whether to transfer land in 2024: No = 0; Yes = 1	0.1915	0.3938
	Credit acquisition	*loan*	Whether to apply for loans from financial institutions in 2024: Expected loan amount not obtained = 0; Expected loan amount = 1	0.1444	0.3518
	Labor allocation	*labor*	The ratio of the number of people engaged in non-agricultural labor in a household to the total population of the household	0.3830	0.2291
Instrumental variable	Promotion efforts for agricultural insurance	*publi*	What do you think of the promotion of agricultural insurance policies? Very small = 1; Small = 2; Generally = 3; Larger = 4; Very large = 5	2.9066	0.8075

### 4.3 Model construction

1 Tobit model. The dependent variable in this article is the income relative deprivation index, which ranges from 0 to 1 and is a restricted variable. Therefore, this article draws on the research of Qian and Li (2020) [36] and uses the Tobit model to verify the impact of agricultural insurance on income inequality among farmers, which can avoid biased and inconsistent parameter estimates. The benchmark model is constructed as follows:

$$\begin{array}{c} y_{i}=\alpha_{\text{1}}+\beta_{\text{1}}{insur}_{i}+\sum_{j=\text{1}}^{n}\theta_{j}z_{ji}+\epsilon_{i} \end{array}$$ (2)

In the above equation, *y* is the dependent variable, namely the Kawani Relative Income Deprivation Index, which represents the degree of income inequality within farmers; *Insur* represents the core explanatory variable, which is whether farmers have purchased agricultural insurance; *α* is a constant term; *Z* represents each control variable; *β*, *θ* is the parameter to be estimated; *ε* is a random interference term.

2 Mediation effect model. Due to the exploration of the role mechanism of production factor allocation in reducing income inequality among farmers through agricultural insurance from three aspects: farmland transfer, credit acquisition, and labor resource allocation, this article draws on the research of Wen et al. (2022) [37], constructs a multiple parallel mediation effect model based on the benchmark regression model, and uses stepwise regression to explore the impact mechanism of agricultural insurance on income inequality within farmers. The specific model construction is as follows:

$$\begin{array}{c} m_{ki}=\alpha_{\text{2}}+\beta_{\text{2}}{insur}_{i}+\sum_{j=\text{1}}^{n}\theta_{j}^{{}^{\prime }}z_{ji}+\epsilon_{i}^{{}^{\prime }} \end{array}$$ (3)

$$\begin{array}{c} y_{i}=\alpha_{\text{3}}+\beta_{\text{1}}^{{}^{\prime }}{insur}_{i}+\sum_{k=\text{1}}^{\text{3}}\gamma_{k}m_{ki}+\sum_{j=\text{1}}^{n}\theta_{j}^{{}^{\prime \prime }}z_{ji}+\epsilon_{i}^{{}^{\prime \prime }} \end{array}$$ (4)

In equations (3) to (4), *m* is the three mediating variables selected in this article, namely agricultural land transfer, credit acquisition, and non-agricultural employment; *γ* is the parameter to be estimated, and the other variables are the same as equation (2). According to the stepwise regression method for testing the mediating effect, firstly, the overall effect of agricultural insurance on income inequality among farmers is verified through formula (2); secondly, in the case of estimation coefficients $\beta_{\text{1}}$ being significant, the impact of agricultural insurance on mediating variables is explored through formula (3); finally, according to formula (4), the mechanism of the mediating variable in the process of agricultural insurance affecting income inequality among farmers is verified, and if $\beta_{\text{1}}^{{}^{\prime }}$ is significant, it indicates that the mediating variable plays a mediating role.

## 5. Empirical results analysis

### 5.1 Benchmark regression analysis of the impact of agricultural insurance on income inequality among farmers

Based on the theoretical analysis in the previous text, this article verifies the impact of agricultural insurance on income inequality among farmers by constructing a Tobit model. The parameter estimation results are shown in Table 3. Among them, columns (1) to (3) show the empirical results of different levels of control variables, including those without control variables, those with individual and household characteristics of household heads, and those with agricultural production characteristics. From Table 3, it can be seen that in the process of gradually adding various control variables, the estimated coefficients of agricultural insurance are all negatively significant at the 1% significance level, indicating that purchasing agricultural insurance can effectively alleviate income inequality among farmers. After adding all control variables, the estimated coefficient of agricultural insurance is −0.0638, indicating that purchasing agricultural insurance can reduce the relative deprivation index of farmers’ income by 0.0638. Hypothesis 1 has been validated.

**Table 3:** Benchmark regression results.

	(1)	(2)	(3)
	Kawani	Kawani	Kawani
*insur*	−0.0568***	−0.0550***	−0.0638***
	(−2.85)	(−2.79)	(−3.24)
*gender*		−0.0112	−0.0041
		(−0.55)	(−0.20)
*age*		0.0002	0.0005
		(0.23)	(0.70)
*edu*		−0.0423***	−0.0467***
		(−3.69)	(−4.04)
*poli*		0.0238	0.0345
		(0.94)	(1.35)
*heal*		−0.0119	−0.0150
		(−0.92)	(−1.12)
*popu*		−0.0300***	−0.0277***
		(−4.91)	(−4.34)
*inawar*		−0.0009	−0.0065
		(−0.04)	(−0.26)
*train*			−0.0397
			(−1.64)
*coop*			−0.0657***
			(−3.03)
*frag*			0.0055***
			(2.63)
*land*			−0.0015*
			(−1.89)
*cons*	0.3905***	0.6572***	0.6882***
	(30.83)	(8.30)	(8.22)
N	632	632	632

Note: * * *, * *, * in the table indicate that the estimated coefficients are significant at the significance levels of 1%, 5%, and 10%, respectively. The values in parentheses are t-values.

In addition, in the regression results of controlling variables, the education level of the head of the household, the number of household members, the acceptance of agricultural technology training, joining agricultural professional cooperatives, and the scale of land management all have a negative and significant impact on income inequality among farmers, while the degree of land fragmentation has a positive impact on income inequality among farmers at a significance level of 5%. That is, the higher the degree of land fragmentation, the more severe the internal income inequality among farmers. The possible reason is that farmers with a high degree of land fragmentation cannot fully enjoy the advantages brought by land scale, mechanized production, and specialized division of labor, resulting in limited growth in household operating income and further widening the income gap with large-scale operating farmers [38].

### 5.2 Robustness test

1 Endogenous processing. As analyzed earlier, purchasing agricultural insurance for farmers can increase their income level and have a greater impact on low-income farmers, which is conducive to narrowing the income gap among farmers. However, the level of income may, in turn, affect the willingness and ability of farmers to purchase agricultural insurance, which may result in endogeneity issues arising from mutual causality in the benchmark model mentioned above. Therefore, this article selects the agricultural insurance promotion intensity (*public*) of insurance companies as an instrumental variable for endogeneity testing. Previous studies have shown that the promotion of agricultural insurance by insurance companies can significantly increase the insurance coverage rate of farmers [39], but it does not have a direct impact on the income level of farmers, which meets the requirements for selecting instrumental variables. The test results are shown in Table 4. According to Table 4, the F-value of the first stage regression is greater than 10, indicating a strong correlation between the selected instrumental variables and endogenous variables, and there is no weak instrumental variable. The regression results of the second stage indicate that purchasing agricultural insurance has a negative impact on income inequality among farmers at a significance level of 5%. Overall, it can be concluded that purchasing agricultural insurance for farmers can significantly reduce the income gap within the household.

**Table 4:** Results of endogeneity test.

	(1)	(2)
	insur	Kawani
*publi*	0.1832***	
	(8.19)	
*insur*		−0.1531**
		(−2.37)
*gender*	0.1813***	0.0124
	(4.75)	(0.52)
*age*	0.0028*	0.0008
	(1.96)	(1.03)
*edu*	0.0146	−0.0443***
	(0.66)	(−3.73)
*poli*	−0.0468	0.0315
	(−0.95)	(1.20)
*heal*	−0.0688***	−0.0215
	(−2.69)	(−1.51)
*popu*	−0.0010	−0.0275***
	(−0.08)	(−4.23)
*inawar*	−0.0749	−0.0163
	(−1.54)	(−0.62)
*train*	−0.0203	−0.0403
	(−0.44)	(−1.63)
*coop*	−0.0776*	−0.0752***
	(−1.87)	(−3.26)
*frag*	0.0069*	0.0063***
	(1.69)	(2.83)
*land*	−0.0012	−0.0016**
	(−0.75)	(−2.00)
*cons*	−0.0287	0.7287***
	(−0.17)	(8.14)
N	632	632
F	10.40

2 Robustness test. This paper uses three methods for robustness testing to verify the robustness of the benchmark regression results mentioned above. (1) Replace the estimation method. This article uses the OLS model to reestimate the impact of purchasing agricultural insurance on income inequality among farmers. (2) Change the sample size. This article adopts the method of sub-sample regression, randomly deleting 10% of the samples and using the remaining samples for regression again. (3) Replace the dependent variable. This article draws on the research of Liu(2023) [5] and recalculates the degree of income inequality among farmers based on the Yitzhak and Podder indices. To avoid dimensional inconsistency caused by tremendous values, this article logarithmizes the Yitzhak index as follows:

$$\begin{array}{c} \text{Yitzhak}=\frac{\text{1}}{n}\sum_{i=k+\text{1}}^{n}\left(x_{i}-x_{k}\right)=\gamma_{{\text{x}}_{k}}^{+}(\mu_{x_{k}}^{+}-x_{k}) \end{array}$$ (5)

$$\begin{array}{c} \text{Podder}=\frac{\text{1}}{n}\sum_{i=k+\text{1}}^{n}\left(lnx_{i}-lnx_{k}\right)=\gamma_{x_{k}}^{+}(\mu_{x_{k}}^{+}-lnx_{k}) \end{array}$$ (6)

According to the regression results in Table 5, the first and second columns show the robustness test results for changing the estimation method and sample size, respectively. The agricultural insurance estimation coefficient is negatively significant at the 1% significance level, and the size, significance level, and impact direction of the estimation coefficient are basically consistent with the benchmark results; The third and fourth columns show the regression results with the replacement of the dependent variable, indicating that purchasing agricultural insurance still hurts income inequality within farmers at a significance level of 1%. In summary, the three robustness tests’ results indicate that participating in agricultural insurance helps alleviate income inequality among farmers, verifying hypothesis 1.

**Table 5:** Results of robustness test.

	(1)	(2)	(3)	(4)
	Change estimation method	Change the number of samples	Yitzhak	Podder
*insur*	−0.0638***	−0.0735***	−0.5051***	−0.1128***
	(−3.21)	(−3.53)	(−3.22)	(−3.01)
*gender*	−0.0041	−0.0075	−0.0333	−0.0377
	(−0.20)	(−0.35)	(−0.21)	(−0.98)
*age*	0.0005	0.0008	0.0042	0.0018
	(0.69)	(0.99)	(0.69)	(1.25)
*edu*	−0.0467***	−0.0428***	−0.3681***	−0.1065***
	(−4.00)	(−3.54)	(−3.99)	(−4.84)
*poli*	0.0345	0.0272	0.2728	0.0800
	(1.33)	(0.97)	(1.33)	(1.63)
*heal*	−0.0150	−0.0228	−0.1188	0.0154
	(−1.11)	(−1.61)	(−1.11)	(0.60)
*popu*	−0.0277***	−0.0310***	−0.2191***	−0.0544***
	(−4.29)	(−4.55)	(−4.29)	(−4.46)
*train*	−0.0397	−0.0411	−0.3138	−0.0781*
	(−1.62)	(−1.61)	(−1.62)	(−1.69)
*coop*	−0.0657***	−0.0566**	−0.5186***	−0.1414***
	(−3.00)	(−2.50)	(−3.00)	(−3.43)
*frag*	0.0055***	0.0047**	0.0438***	0.0076*
	(2.60)	(2.19)	(2.60)	(1.88)
*land*	−0.0015*	−0.0012	−0.0120*	−0.0011
	(−1.87)	(−1.45)	(−1.87)	(−0.69)
*inawar*	−0.0065	−0.0144	−0.0521	0.0214
	(−0.26)	(−0.53)	(−0.26)	(0.44)
*cons*	0.6882***	0.7216***	5.4385***	0.8540***
	(8.13)	(8.08)	(8.13)	(5.35)
R^2^	0.1155	—	0.1155	0.1312
N	632	569	632	632

### 5.3 Analysis of the mediating effect of element configuration

To explore the mechanism pathway through which agricultural insurance affects farmers’ income inequality by optimizing factor allocation (farmland transfer, credit acquisition, and labor allocation), this paper firstly verifies the impact of agricultural insurance on agricultural production factor allocation using the stepwise regression method of mediation effect test, as shown in columns (1) to (3) of Table 6; secondly, it analyzes the impact of factor allocation on income inequality among farmers, as shown in the test results presented in column (4).

**Table 6:** Results of mediation effect test.

	(1)	(2)	(3)	(4)
	*tran*	*loan*	*labor*	Kawani
*insur*	0.4836**	0.4547*	0.0533***	−0.0470**
	(2.21)	(1.86)	(2.95)	(−2.41)
*tran*				−0.0416*
				(−1.76)
*loan*				−0.0873***
				(−3.28)
*labor*				−0.1664***
				(−3.92)
*gender*	0.1341	0.8324***	−0.0012	0.0040
	(0.59)	(2.92)	(−0.06)	(0.20)
*age*	0.0054	0.0062	−0.0001	0.0006
	(0.67)	(0.65)	(−0.11)	(0.83)
*edu*	0.3175**	−0.0824	0.0284***	−0.0407***
	(2.35)	(−0.58)	(2.68)	(−3.56)
*poli*	0.2429	0.2633	0.0687***	0.0502**
	(0.95)	(0.87)	(2.92)	(1.98)
*heal*	−0.1509	0.3095*	−0.0464***	−0.0204
	(−0.95)	(1.75)	(−3.79)	(−1.54)
*popu*	0.0889	−0.0747	0.0158***	−0.0254***
	(1.17)	(−0.96)	(2.70)	(−4.03)
*train*	−0.2388	−0.4787	0.0218	−0.0423*
	(−0.93)	(−1.60)	(0.98)	(−1.78)
*coop*	0.4121*	−0.9437***	−0.0210	−0.0745***
	(1.76)	(−2.90)	(−1.06)	(−3.49)
*frag*	0.0501**	0.0279	−0.0019	0.0060***
	(2.17)	(1.36)	(−0.97)	(2.87)
*land*	−0.0249**	0.0087	0.0005	−0.0015*
	(−2.48)	(1.08)	(0.73)	(−1.83)
*inawar*	0.3885	−0.2389	0.0046	−0.0058
	(1.30)	(−0.78)	(0.20)	(−0.24)
*cons*	−3.2386***	−3.2267***	0.2958***	0.7333***
	(−3.54)	(−3.00)	(3.85)	(8.85)
N	632	632	632	632

(1) The effect of farmland transfer. From column (1) of Table 6, it can be seen that the estimated coefficient of agricultural insurance is 0.4836, which is positively significant at the 5% significance level. This indicates that, under other unchanged conditions, farmers who have not purchased agricultural insurance have a higher probability of transferring land by 0.4836 units than farmers who have purchased agricultural insurance. This empirical result is consistent with the previous expectations, that is, purchasing agricultural insurance stabilises farmers’ income expectations and motivates them to transfer to land to expand production scale. According to the regression results in column (4) of Table 6, the estimated coefficient of farmland transfer is −0.0416, suppressing income inequality within farmers at a significance level of 10%. This indicates that farmers who purchase agricultural insurance can reduce their relative income deprivation index by 0.0416 by transferring land and expanding their scale. As analysed earlier, compared to high-income farmers, the transfer of agricultural land can effectively improve the marginal output level of low-income farmers, thereby increasing their income level and narrowing the income gap. Based on the above analysis, the transfer of agricultural land plays a mediating role in the impact of agricultural insurance on income inequality among farmers, and hypothesis 2−1 is validated.
(2) Credit acquisition effect. According to the regression results in column (2) of Table 6, agricultural insurance can significantly and positively affect farmers’ credit access, with an estimated coefficient of 0.4547, which is significant at the 10% significance level. This indicates that purchasing agricultural insurance can increase the probability of farmers obtaining credit by 0.4547 units. Based on the previous analysis, after purchasing agricultural insurance, farmers can protect agricultural production risks, reduce income fluctuations, and further enhance their ability to repay loans, thereby significantly increasing the quantity and scale of credit support they receive. According to the regression results in column (4) of Table 6, the estimated coefficient of farmers’ credit acquisition is −0.0873, which significantly suppresses income inequality among farmers at the 1% significance level. This indicates that purchasing agricultural insurance can alleviate credit constraints and reduce the relative deprivation index of farmers’ income by 0.0873. After obtaining credit, farmers alleviate liquidity constraints in agricultural production and operation, which helps expand production scale, improve agricultural production efficiency, and increase agricultural operating income. Based on the above analysis, according to the stepwise regression method of the mediation effect test, the credit acquisition of farmers plays a mediating role in agricultural insurance, affecting income inequality among farmers, and hypothesis 2−2 is verified.
(3) Labor force allocation effect. From column (3) of Table 6, it can be seen that the estimated coefficient of agricultural insurance is 0.0533, which positively impacts the labor allocation of rural households at a significance level of 1%. This indicates that, under other unchanged conditions, purchasing agricultural insurance can increase the proportion of non-agricultural labor in rural households by 5.33%. After purchasing agricultural insurance, farmers can diversify their agricultural production risks, promote the adoption of new seeds and technologies, improve output efficiency, save more labor, and encourage them to invest in non-agricultural sectors. According to the regression results in column (4) of Table 6, the estimated coefficient of labor allocation is −0.1664, which significantly suppresses income inequality among farmers at the 1% significance level and can reduce the relative income deprivation index of farmers who have purchased agricultural insurance by 0.1664. After obtaining more non-agricultural employment opportunities, low-income farmers experienced rapid growth in their wage income, narrowing the income gap with high-income farmers. Based on the above analysis, labor allocation plays a mediating role in the impact of agricultural insurance on income inequality among farmers, and hypotheses 2–3 have been validated.

### 5.4 Heterogeneity analysis from the perspective of family life cycle

At various stages of the family life cycle, due to differences in the age structure, family needs, and development tasks of family members, there are also differences in their agricultural production decisions [40]. That is, the family life cycle is closely related to economic behaviors such as labor supply and family income, which affects the allocation of family production factors and ultimately manifests as differences in farmers’ income levels. This article refers to the division of the family life cycle by Weng and Huo (2024) [41] and divides rural households into five stages based on whether there are grandchildren under the age of 16 and elderly people over the age of 65 in the family, as shown in Table 7.

**Table 7:** Basis for dividing family life cycle.

Family life cycle stages	Classification basis
Period of custody	The youngest (grandson) child is under 16 years old, and there are no elderly people over 65 years old
Burden period	The youngest (grandson) child is under 16 years old, and there are elderly people over 65 years old
Stationary phase	The youngest (grandson) child is over 16 years old and there are no elderly people over 65 years old
Maintenance period	The youngest (grandson) child is over 16 years old, and there are elderly people over 65 years old
Empty nest period	There are only elderly people over 65 years old in the family

From the various stages of the family life cycle, firstly, the labor force of farmers in the custody period is relatively young, more receptive to new things and ideas, and has a higher market awareness. Therefore, they are more inclined to expand production scale, adopt new technologies, and other means to increase agricultural income. However, the relatively limited capital accumulation of farmers in the custody period restricts their scale of operation [42]. At this point, purchasing agricultural insurance can provide risk protection for agrarian production and stabilize farmers’ income expectations. Moreover, purchasing agricultural insurance can help farmers obtain credit support, alleviate the dilemma of limited family capital accumulation and inability to expand business scale, and improve farmers’ income levels. Secondly, although the capital accumulation of rural households in the burden period has increased compared to the custody period, they face the dual pressure of custody and support obligations. The number of effective labor force in the family is relatively scarce or even insufficient, and expanding business scale and applying for credit may face significant risks [43]. After purchasing agricultural insurance, most families still rely on increasing working hours to improve their income levels, so the function of optimizing resource allocation in agrarian insurance cannot be efficiently utilized.

Again, for stable farming households, the family’s youngest (grandson) child is over 16 years old, and there are no older adults over 65 years old. The family has a more effective labor force and a smaller support burden. Farmers are more inclined to expand their business scale or engage in non-agricultural employment [44]. Purchasing agricultural insurance further provides risk protection for farmers’ agricultural production and operation, promoting the transfer of surplus labor to non-agricultural sectors and further improving farmers’ income levels. Secondly, rural households in the period of support have higher capital accumulation, lower dependence on external financing channels, and a relatively sufficient labor force. Insurance generally only plays an auxiliary role, so the effect of purchasing agricultural insurance on alleviating income inequality among rural households during the support period is relatively small. Finally, rural households have entered the empty nest stage, with only older adults over 65 in the family, and their physical health has declined [45]. They also have limited time and energy to engage in agricultural production, making non-agricultural employment difficult and leading to high risk aversion. Their acceptance of agricultural insurance, new agricultural technologies, and other things is relatively low. Therefore, the impact of purchasing agricultural insurance on the income of empty-nest households is minimal. Overall, there are differences in the impact of purchasing agricultural insurance on income inequality among farmers at different stages of their family life cycle.

According to the survey sample, there are only 36 households in the empty nest period, which is less representative. Therefore, this article only analyzes the heterogeneity of reducing income inequality within farmers by purchasing agricultural insurance for the remaining four stages of household insurance. The estimated results are shown in Table 8. According to Table 8, significant differences exist in the impact of purchasing agricultural insurance on income inequality among households at different life cycle stages. Agricultural insurance can significantly alleviate income inequality among families in the dependent and stable periods, but it has no significant impact on households at other life cycle stages. When the household is in the period of dependency, the estimated coefficient of agricultural insurance is −0.1038, which can reduce the relative income deprivation index of farmers by 0.1038 at a significance level of 5%, and the inhibitory effect is most significant; When the household of farmers is in a stable period, the estimated coefficient of agricultural insurance is −0.0897, which can reduce the relative income deprivation index of farmers by 0.0897 at a significance level of 5%, verifying the theoretical analysis in the previous text.

**Table 8:** Heterogeneity test results.

	(1)	(2)	(3)	(4)
	Period of custody	Burden period	stationary phase	Maintenance period
*insur*	−0.1038**	0.0260	−0.0897**	−0.0290
	(−2.04)	(0.83)	(−2.46)	(−0.87)
*gender*	−0.0362	−0.0434	0.0240	0.0068
	(−0.62)	(−1.34)	(0.64)	(0.20)
*age*	−0.0012	−0.0013	−0.0014	−0.0016
	(−0.44)	(−1.23)	(−0.60)	(−0.84)
*edu*	−0.2381***	−0.0556***	0.0704**	−0.0405*
	(−3.00)	(−3.52)	(2.53)	(−1.88)
*poli*	0.5369***	0.0782*	−0.0498	−0.2722***
	(3.59)	(1.90)	(−1.32)	(−3.15)
*heal*	−0.0109	−0.0005	−0.0884***	−0.0079
	(−0.22)	(−0.03)	(−3.45)	(−0.29)
*popu*	−0.1423**	0.0202*	−0.0021	−0.1339***
	(−2.18)	(1.83)	(−0.13)	(−6.93)
*train*	−0.0156	−0.0466	0.1995***	−0.1524***
	(−0.17)	(−1.15)	(4.07)	(−4.13)
*coop*	−0.1643**	−0.0180	0.1182**	−0.0864**
	(−2.50)	(−0.56)	(2.45)	(−2.33)
*frag*	0.1644***	0.0031	0.0283	0.0160**
	(4.56)	(1.23)	(1.46)	(2.09)
*land*	−0.0376***	−0.0009	−0.0068*	−0.0094***
	(−4.28)	(−0.88)	(−1.78)	(−3.21)
*inawar*	0.3513**	0.0178	0.0542	−0.2264***
	(2.02)	(0.46)	(1.23)	(−3.76)
*cons*	0.9044***	0.3920***	0.4095*	1.7863***
	(3.01)	(3.13)	(1.91)	(6.78)
N	72	268	140	116

## 6. Conclusion, discussion, and policy implications

### 6.1 Conclusion

This article uses survey data from 632 households in Shandong Province to empirically analyze the impact and mechanism of agricultural insurance on internal income inequality among farmers. The following research conclusions are drawn: (1) Purchasing agricultural insurance can suppress internal income inequality among farmers, and this conclusion still holds after robustness testing and endogeneity processing; (2) Purchasing agricultural insurance can suppress income inequality within farmers through three paths: promoting farmers to transfer to land, alleviating credit constraints, and optimizing the allocation of labor resources for farmers; (3) Purchasing agricultural insurance has a significant inhibitory effect on income inequality among households in the dependency and stable periods, but has no significant impact on households in other stages of their life cycle.

### 6.2 Discussion

Actively expanding channels for increasing farmers’ income and finding endogenous driving forces to narrow the income gap among farmers are essential issues for achieving common prosperity in rural areas. Agricultural insurance is a critical agrarian risk management tool that plays a crucial role in stabilizing farmers’ income. Still, there is little literature on whether agricultural insurance can narrow the income gap among farmers. Based on previous research, this article takes the allocation of production factors as the starting point, focusing on the impact and mechanism of agricultural insurance on the income gap among farmers, as well as the heterogeneity characteristics from the perspective of the life cycle of farmers. It broadens the research scope of rural common prosperity and is a valuable supplement to existing literature.

This study found that purchasing agricultural insurance can effectively alleviate income inequality among farmers. The reasons for this may be income redistribution, post-disaster insurance compensation, and the production incentive effects of agricultural insurance, which promote stable income growth for farmers. Moreover, this promoting effect is more pronounced for low-income farmers, thus narrowing the income gap among farmers. From a mechanism analysis perspective, purchasing agricultural insurance can encourage farmers to optimize the allocation of production factors such as land, funds, and labor. Shandong Province is a central agricultural province, with the country’s total agricultural output value ranking first. It is also an experimental zone for large-scale and intensive development of agriculture. The promotion of agricultural insurance effectively reduces the risks of large-scale operations, concentrates land resources on high-efficiency producers, and can alleviate income gaps caused by differences in business scale; In addition, in Shandong Province, the “policy pledge” of agricultural insurance has eased the credit constraints of low – and middle-income farmers, helped them expand their production scale, introduce new technologies and equipment, and further improve production efficiency. From the results of the heterogeneity analysis, it can be seen that purchasing agricultural insurance significantly inhibits income inequality among farmers in the dependent and stable periods. Families in the custody period face substantial financial pressure and a high degree of risk aversion; therefore, the marginal utility of insurance is maximized. Stable households tend to expand their production scale, and the risk protection role of agricultural insurance can play an important role. The above findings provide crucial theoretical support for improving agricultural insurance product design and enhancing agricultural insurance policy efficiency.

The above research results provide valuable references for efficiently utilizing the agricultural insurance’s rural common prosperity effect. However, there are still shortcomings and room for improvement in the following areas: (1) limitations in the research region. Although this article takes Shandong Province, a central agricultural province in China, as an example to explore the impact and mechanism of agrarian insurance on income inequality among farmers, there is a lack of research on other provinces in China. In this regard, future research can appropriately expand the research area and further study the heterogeneity among Chinese provinces based on their natural resource endowments, agricultural risk levels, and other characteristics. (2) The singularity of research perspective. In the field of agricultural insurance, the insured behavior and insurance coverage level of farmers will have a significant impact on their production behavior and income level. In the future, insurance coverage level will be further included in the research scope.

### 6.3 Policy implications

Based on the above research conclusions, this article obtains the following policy implications: firstly, fully leverage the direct role of agricultural insurance in stabilizing income and increasing income, and narrowing the income gap among farmers. According to research data, the current agricultural insurance coverage rate is generally at a low level, which is mainly due to insufficient supply capacity of other crop insurance products except for grain crop insurance, and farmers are unable to obtain corresponding insurance services. Therefore, it is proposed to continue to promote the “enhancement” of agricultural insurance and increase the publicity of agricultural insurance to improve the insurance coverage rate of farmers. Secondly, promote the coordinated cooperation between agricultural insurance, land, capital, and non-agricultural employment policies, so as to facilitate the efficient allocation of production factor resources among farmers. On the one hand, enhancing the coordination and cooperation between agricultural insurance and financial instruments, exploring the implementation of the “agricultural insurance+package of financial products” guarantee project, and promoting the integration of farmers and rural financial markets; on the other hand, promoting the large-scale development of agriculture, appropriately increasing the support of agricultural insurance for large-scale farmers, providing relatively favorable insurance rates and higher levels of risk protection. The third is to promote the differentiation of agricultural insurance products, so that farmers at different stages of their family life cycle can choose suitable insurance products according to their needs, enhance their risk tolerance, unleash the development potential of farmers’ families, and achieve the effect of optimizing production factor allocation and improving income levels.
